# Tracking biomass burning markers in atmospheric aerosols: Robust quantification of anhydrosugars and reference material evaluation

**DOI:** 10.1007/s11356-026-38136-6

**Published:** 2026-08-11

**Authors:** Rosa Pérez-Pastor, Susana García-Alonso, María Leco, Óscar Pindado

**Affiliations:** Chromatography Unit, Technology Department, CIEMAT, Av. Complutense, 40, Madrid, 28040 Spain

**Keywords:** Levoglucosan, Biomass burning tracers, Atmospheric particulate matter, Source apportionment, Quality assurance, Measurement uncertainty estimation

## Abstract

Biomass burning significantly impacts air quality and regional climate processes, making the precise quantification of its molecular tracers critical for source apportionment models. To ensure regional data comparability, establishing robust and standardized analytical frameworks is essential. This study presents a validated method for determining key biomass burning tracers: levoglucosan, galactosan, and mannosan. Accelerated solvent extraction (ASE) with a dichloromethane:methanol mixture was selected as the extraction procedure, due to its high precision, automation and minimized sample handling, followed by BSTFA derivatization and GC/MS. The method showed high linearity (*R*^2^ > 0.9987), repeatability below 6%, and limits of detection lower than 0.02 mg L⁻^1^. Accuracy was evaluated using SRM 2787 (Fine particulate matter), yielding relative errors between 1% and 7.4%. In contrast, significant variability was observed for levoglucosan concentrations in SRM 1649b (Urban dust), depending on extraction conditions, highlighting the strong influence of matrix aging and methodological factors on older reference materials. Chromatographic calibration was identified as the dominant contribution to the calculated uncertainty budget. The resulting expanded uncertainties fully comply with upcoming European air quality monitoring directives.

**Supplementary information:**

The online version contains supplementary material available at 10.1007/s11356-026-38136-6.

## Introduction

Understanding the abundance, composition, and sources of organic compounds in aerosols is crucial for mitigating their adverse impacts on both the atmospheric environment and human health (Janoszka et al. 2020; Zhu et al. 2022). Several studies have shown that measures aimed at reducing air pollutants, mainly focused on reducing traffic emissions in urban areas, have not provided the expected results. These abatement strategies did not consider pollutants from sources such as biomass burning, due to the lack of information on their magnitude and origin (Titos et al. 2017).

Biomass burning significantly impacts regional and global climate processes, yet its contribution is often underestimated (Li et al. 2021; Schkolnik and Rudich 2006). To address this, levoglucosan and its isomers, generated during the thermal decomposition of cellulose and hemicellulose at temperatures above 300 °C, have emerged as key molecular tracers, as they are uniquely produced during wood combustion (Simoneit et al. 1999). Levoglucosan is emitted in large quantities, allowing it to be detected far from the emission source (Elias et al. 2001; Kuo et al. 2008; Louchouarn et al. 2009). Although some studies have questioned their general use for apportionment of aged biomass burning emissions (Hoffmann et al. 2010; Li et al. 2021), these compounds are widely accepted as biomass burning tracers. The relative abundance of levoglucosan and its isomers, particularly the levoglucosan to mannosan (L/M) ratio, is often used for discriminating specific combustion sources (Marynowski and Simoneit 2022; Vicente and Alves 2018), allowing us to distinguish between softwoods, hardwoods, and agricultural waste (Janoszka et al. 2020; Louchouarn et al. 2009; Schmidl et al. 2011). However, the use of these ratios may be limited, particularly for long-range transport studies, due to the significant degradation that also isomers can undergo.

Extensive research has been conducted in recent years on the determination of anhydrosugars in atmospheric samples (Bhattarai et al. 2019; Schkolnik and Rudich 2006). Levoglucosan has been included in multiple source apportionment studies (Yttri et al. 2021). Its ratio to organic carbon has been used to estimate the relative contribution of biomass burning in wood smoke emissions (Bikkina et al. 2022; Cheng et al. 2013; Maenhaut et al. 2012; Puxbaum et al. 2007). Additionally, a strong correlation has been observed between levoglucosan and PAHs (Monteiro et al. 2018), whereas no correlation was identified with traffic markers such as hopanes (Jedynska et al. 2015; Saha et al. 2017). In fact, it has recently been proposed as a potential air pollutant to be monitored under future European directives alongside methane, ammonia, and black carbon (Monforti-Ferrario et al. 2022).

The main techniques used for anhydrosugar determination are high-performance anion exchange chromatography coupled with pulsed amperometric detector (HPAEC-PAD), high-performance liquid chromatography coupled with mass spectrometry (HPLC/MS) and gas chromatography coupled to mass spectrometry (GC/MS) (Janoszka and Czaplicka 2019; Piot et al. 2012). Therefore, the development and application of precise chromatographic techniques is essential to ensure accurate quantification so as to mitigate uncertainties in source apportionment models.

Previous intercomparison exercises have demonstrated that, although acceptable agreement can generally be achieved for anhydrosugar determination, methodological variability remains an important source of uncertainty (Verlhac 2013; Yttri et al. 2015). Among all the available techniques to overcome the analytical difficulties of quantifying levoglucosan, GC/MS stands out due to its higher sensitivity, its ability to separate the three isomers, and its capacity to simultaneously analyze other compounds of interest, beyond sugar markers, for particulate matter characterisation.

Efforts to establish standardized extraction protocols, solvent selection, and quantification methods for GC/MS analysis are ongoing, although only a limited number of fully validated procedures are currently available (Cordell et al. 2014; Louchouarn et al. 2009; Parshintsev and Hyötyläinen 2015). Recently, the CEN Technical Committee on Air Quality has published a standardized method for levoglucosan determination in PM, based on either HPAEC-PAD or GC/MS (European Committee for Standardization 2024).

Several certified reference materials are available for atmospheric aerosol particles, such as SRM 1648a (urban PM), SRM1649b (urban dust), and SRM 2787 (fine PM < 10 μm). However, most of these materials are well characterized only for the analysis of PAHs, PCBs, and metals (Wise 2022). Although SRM1649b has been suggested as a potential working standard for levoglucosan analysis (Kuo et al. 2008), the determination of anhydrosugars in that material is significantly complicated by notable matrix effects so lead to a discrepancy between results reported by different authors and a lack of official consensus yet remains (Gao et al. 2018).

This paper presents an effort to develop and optimize the extraction procedure, as anhydrosugars within aged particulate matter may be strongly bound. It is imperative that analytical frameworks incorporate a rigorous assessment of measurement uncertainty to ensure the reliability of the data. Such characterization is vital for refining source apportionment models and improving their predictive accuracy.

To address these ongoing analytical debates, the main objective of this study was to systematically evaluate how different solvents and extraction techniques influence the recovery and reproducibility of anhydrosugars from atmospheric aerosols sampled on quartz filters and determined by GC/MS, focusing on robust validation parameters. Therefore, the present work provides a detailed bottom-up uncertainty study for the determination of levoglucosan and its isomers in particulate matter, identifying the primary sources of variance in the analytical process. Furthermore, by providing a detailed comparative analysis of both SRM 1649b and SRM 2787, this work aims to clarify persistent discrepancies in reference materials, offering new insights into how sample treatment shapes our understanding of biomass burning markers.

## Experimental

### Chemicals and reagents

Anhydrosugars: levoglucosan was purchased from Sigma-Aldrich (*Deisenhofen, Germany*), galactosan and mannosan were obtained from Carbosynth (*Compton, UK*).

Surrogate/Internal standards: Sedoheptulosan and o-terphenyl were from Sigma-Aldrich, (*Deisenhofen, Germany*).

Derivatization reagents: N,O-bis(trimethylsilyl)trifluoroacetamide (BSTFA) containing 1% of trimethylchlorosilane (TMCS) and pyridine were obtained from Sigma-Aldrich. (*Deisenhofen, Germany*).

Solvents: dichloromethane and methanol were supplied by J.T. Baker (*Phillipsburg, N.J.),* while acetonitrile was purchased from Romil (*Cambridge, U.K.*).

Reference materials: SRM1649b was prepared by sieving the bulk SRM 1649 material through a 63 µm mesh to improve homogeneity, which was an urban dust collected in Washington, DC, in 1982. SRM 2787 (fine PM < 10 µm) was developed in 2011 (Schantz et al. 2016). This material was used for method validation, with reference values established by Louchouarn (Louchouarn et al. 2009).

### Aerosol sampling

Twenty-four-hour PM_10_ samples evaluated in this study were collected using a high-volume sampler (Digitel DHA-80) operating at a flow rate of 30 m^3^ h^−1^, within the framework of a previous study (Pérez Pastor et al. 2020).

Samples were collected on 150 mm quartz filters (Pall Tissuquartz™ 2500 QAT-UP), which were subsequently divided into eight portions for further analysis.

### Extraction optimization

Three solvents were evaluated for extraction performance: methanol, a mixture of dichloromethane/methanol (DCM/MeOH) 2:1 v/v, and acetonitrile.

Quartz filter fractions (1/8) were spiked with 50 µL of a standard solution (20 mg L^−1^), corresponding to a final concentration of 1 mg L^−1^, prepared in methanol or acetonitrile and containing levoglucosan, galactosan, mannosan, and sedoheptulosan (used as surrogate standard).

Filters were extracted in triplicate by ultrasonic agitation and subsequently derivatized with BSTFA/TMCS, following a previously reported method (Pérez Pastor et al. 2020).

Recovery was determined by comparison with standards prepared in the same solvent used for extraction (methanol or acetonitrile) and subjected to the same derivatization procedure.

After selection of the optimal extraction solvent, the efficiency of different extraction techniques was evaluated. The study was conducted using both spiked blank filters and real PM samples with high and low anhydrosugar loadings.

A DCM/MeOH mixture (2:1, v/v) was used for the extraction of spiked filters or samples (in triplicate) using the following procedures:

- Sonication: filters were extracted in closed culture tubes with 5 mL solvent mixture for 15 min. The procedure was repeated three times.
- Vortex-assisted sonication: filters were first vortexed with 5 mL of solvent mixture for 30 min, followed by sonication as described above.
- Accelerated solvent extraction: extraction was performed using an ASE 350 (*Dionex, Thermofisher Scientific, Sunnyvale, CA*) with 10 mL stainless-steel cells. Two static cycles were applied at 100 °C and 100 bars (1500 psi), using approximately 10 mL of solvent mixture, for 10 min. Hydromatrix was used as a dispersant to fill void volumes.
- Automated Soxhlet extraction: performed using a Büchi B811 system. 50 mL of solvent mixture was added to the extraction thimble. Condensers were cooled with water maintained at −4 °C, and extraction was carried out for 24 h.

Extracts were filtered through 0.45 µm PTFE membranes and concentrated to 1 mL under a gentle stream of nitrogen.

### Derivatization and GC/MS analysis

Due to the high polarity of the analytes, a derivatization step was required prior to GC/MS analysis. Anhydrosugars were derivatized by trimethylsilylation using BSTFA/TMCS. Several derivatization conditions were tested, including varying volumes of BSTFA (25–50 µL), pyridine (10–45 µL), and isooctane (0–60 µL) as solvent.

Aliquots of 100 µL of the extract were evaporated to dryness. Subsequently, 50 µL of BSTFA, 45 µL of pyridine, and 5 µL of internal standard (o-terphenyl) were added. The reaction was carried out at 70* °C* for 1 h. After cooling, the trimethylsilyl derivatives were analyzed by GC/MS using an Agilent 7890B gas chromatograph coupled to a 5977 A mass spectrometer (*Agilent Technologies, Wokingham, UK*), equipped with a fused silica capillary column HP-5MS-UI (30 m × 0.25 mm i.d. × 0.25 µm film thickness).

Samples (2 µL) were injected in splitless mode. Helium was used as carrier gas (1 mL min^−1^). The GC oven programme was as follows: initial temperature 100 °C, ramped to 200 °C at 15 °C min^−1^ (hold 1 min), then increased to 300 °C at 25° min^−1^ (hold 5 min). The injector and GC/MS interface were set at 250 °C. The ion source temperature was 230 °C and the quadrupole temperature was 150˚C.

The mass spectrometer operated in electron impact mode (70 eV) using selected ion monitoring (SIM). The monitored ions (m/z) were 204, 217, 230 and 333. Mannosan and levoglucosan were quantified using m/z 204; galactosan and sedoheptulosan using m/z 217; o-terphenyl using m/z 230; and m/z 333 was used as a qualifier ion. Compounds were identified by alignment of retention times with authentic standards and also the relative abundance ratios between quantifier and qualifier ions.

## Results and discussion

### Optimization of sample extraction

Various extraction techniques have been successfully applied for anhydrosugar determination, including Soxhlet extraction (Larsen et al. 2006), accelerated solvent extraction (Kuo et al. 2008), and sonication (Janoszka and Czaplicka 2019), the latter being the most commonly utilised technique.

Based on previous studies highlighting the importance of incorporating a polar solvent to promote particle swelling and enhance the extraction of compounds trapped within the internal structure of aerosol particles (Louchouarn et al. 2009), three solvents were evaluated: methanol, acetonitrile and a DCM/MeOH mixture (2:1, v/v).

Triplicate analysis of spiked filters extracted by sonication with each solvent was performed. The recovery percentages obtained for the target anhydrosugars are presented in Table 1.

**Table 1:** Recovery of anhydrosugars from spiked filters using different solvents

	Recovery (%)		
	MeOH	ACN	DCM/MeOH
Galactosan	89	85	84
Mannosan	84	80	91
Levoglucosan	86	76	92
Sedoheptulosan	87	69	90

The DCM/MeOH mixture was selected as the optimal extraction solvent, as it provided better recoveries. In addition, this solvent system offers practical advantages, including easier drying process previous to derivatization step and the possibility of simultaneously determining non-polar compounds, such as PAHs, in the same extract.

**Fig. 1:**
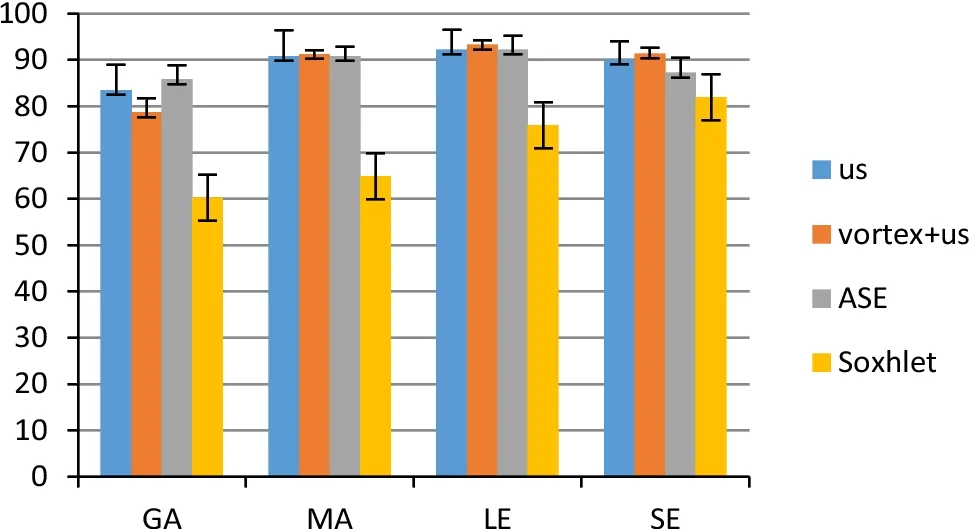
Extraction of anhydrosugars (%) using different extraction techniques (Galactosan (GA), mannosan (MA), levoglucosan (LE), sedoheptulosan (SE))

After selecting the extraction solvent, the efficiency of four extraction techniques—sonication, vortex-assisted sonication, ASE, and Soxhlet extraction—was evaluated using spiked quartz filters. The results are summarized in Fig. 1.

While sonication and ASE achieved satisfactory recoveries (> 80%) within relatively short processing times (15–30 min), Soxhlet extraction, despite being a traditional reference method, yielded consistently lower results for all target compounds. This is likely attributable to the prolonged 24-h extraction cycle, which could favours the analyte degradation. The addition of a vortex step prior to sonication did not significantly improve extraction efficiency; therefore, it was ruled out.

To further assess method performance, real atmospheric samples (Sect. "Aerosol sampling") with both high and low anhydrosugar loadings were analyzed using sonication and ASE. The results are presented in Table 2.

**Table 2:** Results of real samples extracted using ASE and Sonication (ng m^−3^)

	Sample 1		Sample 2		Sample 3		Sample 4	
	US	ASE	US	ASE	US	ASE	US	ASE
Galactosan	275	287	324	365	0.22	0.30	0.16	0.33
Mannosan	272	252	283	278	0.54	0.88	0.65	0.77
Levoglucosan	5437	4892	5826	5949	4.8	7.6	5.9	8.3

Although statistical analysis (Paired sample *t*-test, Statgraphics) revealed no significant differences between the two techniques (P0 = 0.258, *α* = 0.05), indicating that both techniques are suitable for anhydrosugar extraction, ASE was selected as the reference technique for the final protocol. This choice is justified by its superior precision and the high degree of automation, which minimizes manual sample handling, eliminating the post-extraction filtration step, thereby reducing potential sources of analytical error and ensuring high throughput for large-scale monitoring campaigns.

### Derivatization and GC/MS analysis

Trimethylsilylation is the most widely used derivatization approach for anhydrosugars, employing reagents such as BSTFA, BSTFA/TMCS, TMSI-N-trimethylsilylylimidazole, or MSTFA. The efficiency of the derivatization depends on reaction time and temperature, derivatization reagent and solvent used (Janoszka and Czaplicka 2019).

In the study carried out on derivatization with BSTFA/TMCS, the only condition that resulted in significantly lower signals for galactosan and mannosan involved the use of 25 µL BSTFA and 60 µL isooctane. It is important to note the significance of adding pyridine. Experiments have indicated that complete derivatization was only achieved in the presence of pyridine, which acts as both a catalyst and a proton acceptor. Increasing the amount of pyridine resulted in a corresponding increase in signal intensity.

Based on these results, the optimized derivatization procedure involved a 100 µL aliquot of extract under nitrogen, followed by the addition of 50 µL of BSTFA, 45 µL of pyridine and 5 µL of internal standard, as detailed in Sect. "Derivatization and GC/MS analysis".

### Method validation

The analytical performance of the optimized procedure was evaluated in terms of linearity, repeatability, intermediate precision, limits of detection (LOD) and quantification (LOQ), and accuracy. The main validation parameters are summarized in Table 3.

**Table 3:** Validation parameters of the proposed method

	LD (mg L^−1^)	LQ (mg L^−1^)	RSD (%)	Recovery (%)	Intermediate precision
Galactosan	0.01	0.03	4.4	84.0	10.3
Mannosan	0.02	0.06	5.5	90.8	12.0
Levoglucosan	0.02	0.06	5.6	92.2	10.8

#### Linearity

A stock solution (100 mg L^−1^) of each compound (galactosan, mannosan, levoglucosan, and sedoheptulosan as internal standard) was prepared gravimetrically in methanol. Calibration standards were obtained by serial dilution in the range 0.05–30 mg L^−1^. Calibration curves were constructed using the internal standard method, based on triplicate measurements of 8 calibration levels. Aliquots were evaporated and derivatized following the same procedure as for samples.

Linearity was evaluated by least-squares regression. Two concentration ranges (0.05–2 mg L^−1^ and 2–30 mg L^−1^) were established. Good linearity was obtained, with correlation coefficients (R^2^) higher than 0.9987 in all cases.

#### Repeatability

Repeatability was assessed as the relative standard deviation (RSD) for five replicate analyses of spiked filters (1 mg L^−1^) performed on the same day. RSD values ranged from 4.4% for galactosan to 5.6% for levoglucosan.

#### Limits of detection (MDL) and quantification (LOQ)

LOD and LOQ were calculated as three and ten times, respectively, the standard deviation of six replicate analyses of blank filters. Values were below 0.02 mg L^−1^ (LOD) and 0.06 mg L^−1^ (LOQ).

#### Intermediate Precision

Intermediate precision was evaluated by analyzing eight aliquots of SRM1649b (30 mg each, in duplicate) over a 1-year period. RSD values were below 12.0% for all three isomers.

#### Assessment of method performance using Certified Reference Materials

A preliminary assessment of the trueness was conducted using SRM 2787. Due to the limited availability of this material, only one aliquot (30 mg) could be analyzed in triplicate. The results showed satisfactory agreement with certified values (relative errors between 1.0% and 7.4%). This confirms the effectiveness of the optimized procedure for aerosol matrices (Table 4).

**Table 4:** Analysis of SRM2787

	Certified (mg kg^−1^)	Measured (mg kg^−1^)	Relative error (%)
Galactosan	35.3 ± 0.8	32.7	7.4
Mannosan	101 ± 2	102	1.0
Levoglucosan	496 ± 6	486	2.0

### Estimation of uncertainty

Uncertainty was estimated following the ISO bottom-up approach, which involves the identification and evaluation of all relevant sources of uncertainty and their subsequent combination to obtain the overall uncertainty (Ellison and Williams 2012). The procedure comprises four steps: specification of the measurand, identification of sources, quantification of individual components, and calculation of the combined uncertainty.

The first step involves the specification of the measurand. The concentration of the analyte in the aerosol, *C*_a_ (ng m^−3^), was calculated as:

$$C_a=\frac{C_{dis}\;FV_s}{V_aR}1000$$

where *C*_dis_ is the analyte concentration (mg L^−1^) in the measured solution, *F* is the analyzed filter fraction, *V*_s_ is the sample volume (1 mL), *V*_a_ is the sampled air volume, *R* is the recovery, 1000 is a conversion factor (mg to ng).

The second step comprises identification of sources. Five primary sources of uncertainty were identified:

- Chromatographic analysis (uC_dis_), which includes concentration of sample and derivatization step, standard preparation, calibration and repeatability.
- Extract volume (uV_s_)
- Sampled air volume (uV_a_)
- Filter subsampling (uF)
- Method recovery (uR)

The third step involves quantifying each of these components of uncertainty. The procedure followed is briefly described below, and detailed in ESM.

#### Chromatographic analysis (uC_dis_)

The uncertainty associated with chromatographic determination was estimated by combining contributions from (a) standard preparation, which includes the gravimetric preparation of the stock solution (u_Cstk_) and the subsequent dilutions (u_dil_) (b) calibration curve (u_Ccal_) and (c) instrumental repeatability(u_rep_).

$$\left(\frac{u_{Cdis}}{C_{dis}}\right)^2=\left(\frac{u_{Cstk}}{C_{stk}}\right)^2+\left(\frac{u_{dil}}{C_{dil}}\right)^2+\left(\frac{u_{Ccal}}{C_{dis}}\right)^2+\left(\frac{u_{rep}}{C_{dis}}\right)^2$$

Table 5 summarizes uncertainties associated with the analyte concentration in the measured dissolution, *C*_dis_

**Table 5:** Relative uncertainty contributions to *C*_dis_

	$\left(\frac{u_{Cstk}}{C_{stk}}\right)^2$	$\left(\frac{u_{Cdil}}{C_{dil}}\right)^2$	$\left(\frac{u_{Ccal}}{C_{cal}}\right)^2$	$\left(\frac{u_{rep}}C\right)^2$	$\left(\frac{u_{Cdis}}{C_{dis}}\right)^2$	$\frac{u_{Cdis}}{C_{dis}}$
Galactosan	0.0013	0.0001	0.022	4.8 10^–4^	**0.024**	**0.15**
Mannosan	0.0013	0.0001	0.026	3.2 10^–4^	**0.028**	**0.17**
Levoglucosan	0.0013	0.0001	0.015	4.0 10^–4^	**0.017**	**0.13**

The combined uncertainty due to the measured dissolution was dominated by calibration, whereas contributions from dilution and repeatability were minor. This may be due to the derivatization, which is included in this calculation, being the most critical step in the method, by the low volumes used and the relatively high temperatures of reaction.

#### Extract volume (Vf)

Uncertainty in the final extract volume (u_Vf_, 1 mL) arises from syringe tolerance, temperature, and repeatability.

$$\left(\frac{u_{V_f}}{V_f}\right)^2=9.8\;10^{-5}$$

#### Sampled air volume

The uncertainty of the air volume (u_Va_) depends on the flow rate (*Q*) and the time, and is expressed as:

$${\left(\frac{{u}_{Va}}{{V}_{a}}\right)}^{2}={\left(\frac{{u}_{Q}}{Q}\right)}^{2}+{\left(\frac{{u}_{t}}{t}\right)}^{2}$$

$$\frac{{u}_{Va}}{{V}_{a}}=0.036$$

#### Filter subsampling (F)

Uncertainty associated with the analyzed filter fraction (u_VF,_ 1/8) was estimated from replicate weighing (six replicates, *f *= 1.3) of a filter and the corresponding fraction:

$$\frac{u_{VF}}{V_F}=0.036$$

Main contribution comes from the uncertainty of the sampled air, the contribution of subsampling negligible.

#### Recovery (filter extraction and concentration)

Recovery was assessed as a type A uncertainty (u_rR_) using four spiked filters (3 ng mL^−1^). The associated standard deviation was corrected to exclude chromatographic contributions.

#### Combined uncertainty

The final step is to calculate the combined relative uncertainty. It was calculated as follows:

$$\frac{{u}_{Ca}}{{C}_{a}}=\sqrt{{\left(\frac{{u}_{Cdis}}{{C}_{dis}}\right)}^{2}+{\left(\frac{u{V}_{s}}{{V}_{s}}\right)}^{2}+{\left(\frac{{u}_{Va}}{{V}_{a}}\right)}^{2}+{\left(\frac{{ur}_{R}}{R}\right)}^{2}}$$

The resulting values ranged between 14% and 18%, depending on the compound (Table 6).

**Table 6:** Combined and expanded uncertainty

	$\left(\frac{u_{Cdis}}{C_{dis}}\right)^2$	$\left(\frac{uV_s}{V_s}\right)^2$	$\left(\frac{u_{Va}}{V_a}\right)^2$	$\left(\frac{u_R}R\right)^2$	$\left(\frac{u_{Ca}}{C_a}\right)^2$	$\left(\frac{u_{Ca}}{C_a}\right)$	$u_{exp}\left(\%\right)$
Galactosan	0.024	0.0001	0.0013	0.0048	**0.030**	**0.17**	**34**
Mannosan	0.028	0.0001	0.0013	0.0025	**0.032**	**0.18**	**36**
Levoglucosan	0.017	0.0001	0.0013	0.0025	**0.021**	**0.14**	**28**

Expanded uncertainty was calculated using a coverage factor *k *= 2, corresponding to a confidence level of 95% (Table 6).

**Fig. 2:**
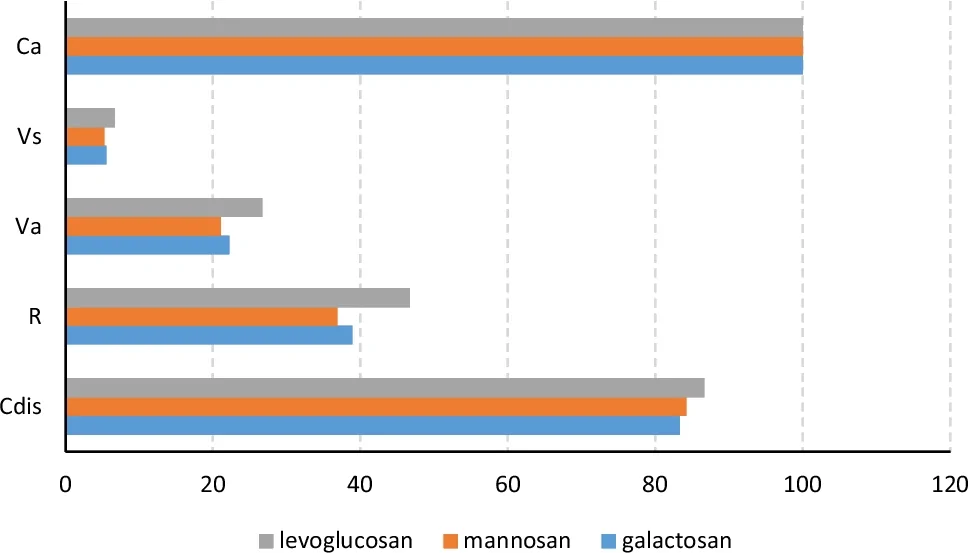
Relative contribution of individual sources to combined uncertainty for the analyzed compounds, expressed as a percentage of total variance. Ca, air concentration; Vs, analyzed sample volume; *V*_a_, sampled air volume; *R*, recovery; *C*_dis_, measurement solution concentration

The expanded uncertainty values (28–36% with *k* = 2) fall within the acceptable ranges for this type of environmental analysis. In fact, they are consistent with, and even better than, the requirements established by the European Directive 2004/107/EC for other pollutants such as benzo(a)pyrene (< 50%) (European Parliament and the Council 2004). A graphical representation of the relative contributions to the combined uncertainty (Fig. 2) shows that chromatographic determination is the dominant source, while contributions from extract volume and dilution are negligible. Secondary contributions arise from recovery and air volume.

The uncertainty budget reveals that the overall uncertainty is primarily driven by the chromatographic determination, particularly the calibration step, which constitutes the dominant contribution to uC_dis_. This behaviour is consistent with the relatively high residual variability observed in the calibration curves, and agrees with previous studies highlighting calibration as a major source of uncertainty in chromatographic methods (Ellison and Williams 2012). This indicates that improvements in calibration strategy (e.g. increasing the number of calibration levels, optimizing the concentration range, or applying weighted regression) could reduce total uncertainty (Miller and Miller 2018). In contrast, contributions from dilution and instrumental repeatability were found to be negligible, confirming the robustness of the sample preparation and analytical procedures. Similar findings have been reported in aerosol chemical analysis, where uncertainties associated with volumetric operations are typically minor compared to those arising from calibration and signal variability, especially when derivatization is involved in the procedure (Jiménez and Pastor 2014).

Among the remaining factors, the uncertainty associated with the sampled air volume represents a moderate contribution, mainly due to flow rate variability inherent to high-volume samplers. This contribution is unavoidable under field sampling conditions and falls within the range reported for similar sampling systems operating under ambient conditions (European Committee for Standardization 2014). The uncertainty associated with recovery also contributes non-negligibly, reflecting variability in extraction efficiency; however, its impact remains lower than that of calibration.

Overall, the combined relative uncertainty ranged between 14% and 18%, which is higher than the intermediate precision obtained for reference materials. This discrepancy highlights the importance of applying a full bottom-up uncertainty approach, as reliance solely on precision metrics may lead to underestimation of total analytical uncertainty (Ellison and Williams 2012), as routine precision estimates may underestimate the true analytical uncertainty. These results indicate that future methodological improvements should prioritize calibration procedures and, to a lesser extent, flow control during sampling, as these are the main limiting factors in achieving lower uncertainty levels.

Finally, it is important to relate this uncertainty calculation to the atmospheric degradation of anhydrosugars. Establishing a well-defined analytical uncertainty for these compounds is essential to ensure that source apportionment models do not confound measurement error with actual chemical loss in the atmosphere, thereby improving the reliability of biomass burning contribution estimates.

### Analysis of SRM1649b

The determination of anhydrosugars, particularly levoglucosan, in SRM 1649b has shown significant variability across laboratories and analytical approaches over the years. This is particularly relevant given that, prior to the availability of certified reference materials such as SRM 2787, SRM 1649b was widely used for this purpose.

The value certified by NIST in 2009 (81.1 ± 6.1 mg kg⁻^1^), based on the work of Larsen et al (2006), is considerably lower than values reported in subsequent studies, which typically range from 150 to 170 mg kg⁻^1^. For instance, Gao et al (2018) proposed an average value of 163 ± 4.5 mg kg⁻^1^ based on previously reported data. Nevertheless, several other laboratories have reported values similar to those obtained by Larsen (Grandesso and Ballesta 2014; Harrison et al. 2012). This systematic difference suggests that the discrepancy is not due to random analytical error alone, but rather to methodological differences, sample treatment, or matrix effects associated with SRM 1649b.

The concentrations of the three anhydrosugar isomers (mg kg⁻^1^), expressed as the mean of three repeated tests, are shown in Table 7. These tests were carried out on 30 mg samples using either ASE or ultrasound extraction. The table also compiles values reported by different laboratories, as well as the mean value obtained at an intercomparison exercise conducted within ACTRIS (Verlhac 2013).

**Table 7:** Literature review of anhydrosugars results for SRM1649b (mg kg^−1^)

Reference	Levoglucosan (RSD)	Galactosan (RSD)	Mannosan (RSD)	Extraction	Technique
*(Larsen III et al.* 2006*)*					
*Ambient temperature*	81.1 (9.4)			PFE/Soxhlet	GC/MS der
*Frozen*	162 (8)				
*(* Kuo et al. 2008*)*	163.9 (118)			PFE	GC/MS der
*(* Louchouarn et al. 2009*)*	160.5 (4.7)	4.8 (0.2)	16.7 (0.7)	PFE	GC/MS der
*(* Brandenberger et al. 2009*)*	139.1 (6.19			PFE	GC/MS der
*(* Orasche et al. 2011*)*	165.0 (1.4)			In situ der TD	GC/MS
*(* Harrison et al. 2012*)*	64.8–89.21			US/water	GC/MS der
*(*Verlhac 2013*)*	153.3	4.6	16		Various
*(* Kirchgeorg et al. 2014*)*	168 (4.5)	5.0 (0.2)	15.7 (0.7)	PFE	IC/MS
*(* Grandesso and Ballesta 2014*)*	85 (11)			TD	GC/MS der
*(* Pomata et al. 2014*)*				PFE	GC/MS der
*Internal standard*	148.54 (18.39)				
*Standard addition 1*	173.23 (27.68)				
*Standard additions 2*	160.3 (16.22)				
*(* Buiarelli et al. 2018*)*					
*Ambient temperature*	90.3 (15.1)			US/water	HPLC/MS/MS
*Frozen*	179.1 (18)				
*(* Gao et al. 2018*)*					
*Internal standard*	171 (6.99)				
*Standard addition 1 (10 mg)*	158 (1.5)			PFE/ethanol	GC/MS der
*Standard additions 2 (30 mg)*	165 (7.16)				
*(* Mashayekhy Rad et al. 2018*)*	152			US/water	HPLC/MS/MS
*(* Stevens et al. 2025*)*	173.9 (16.9)	20.6 (1.2)	9.7 (0.8)	US/water	HPLC/MS/MS
*This work*					
*Ambient temperature*	156 (10.8)	4.7 (0.7)	16.0 (0.8)	PFE	GC/MS der
*ambient temperature*	90.1 (4.7)	4.9 (0.9)	15.5 (1.0)	US	

In this study, ultrasound extraction produced levoglucosan concentrations similar to those reported at the lower range (Buiarelli et al. 2018; Harrison et al. 2012; Larsen III et al. 2006). It should be noted that these results, when specified, were obtained from an SRM material stored at ambient temperature. Similar values were obtained in our laboratory, even after purchasing a new aliquot of the material, suggesting that the results are reproducible and not related to the degradation of a specific batch.

Freezing at very low temperatures, such as −80 °C, is a recognized method of enhancing extraction efficiency by physically disrupting the sample. The higher values obtained by Larsen III et al (2006) and Buiarelli et al (2018) from cryogenically stored samples may therefore be explained by increased extraction efficiency, even in the absence of a polar solvent. These results support the hypothesis that storage conditions may influence extraction efficiency or analyte availability in this material.

The extraction technique and the material used to fill the extraction cell also seem to influence the results. In this study, levoglucosan analysis using ASE with diatomaceous earth as the filling matrix provided a mean value of 139.3 mg kg^−1^, which is consistent with values reported by Brandenberger et al (2009) and Mashayekhy Rad et al (2018). According to these authors, this value represents around 85% of the concentrations previously reported by the same working group, and was explained by the filling matrix used in the ASE cells. They suggest that the diatomaceous earth sorbent may shield fine particles from the solvent, resulting in lower anhydrosugar yields than when they used combusted sand in prior studies (Kuo et al. 2008; Louchouarn et al. 2009).

The calibration methodology may also contribute to variability in the reported concentrations. For instance, the levoglucosan results obtained in this study, which used internal standard calibration, are similar to those reported by Pomata et al (2014) when an internal standard calibration curve was used instead of standard additions, which resulted in higher concentration values. In contrast, Gao et al (2018) observed a decrease in levoglucosan concentration when standard addition calibration was applied.

These differences may be related to matrix effects, which are known to be significant in complex particulate matter matrices such as SRM 1649b. The composition of this complex matrix may affect analyte recovery, extraction efficiency, and instrumental response, leading to method-dependent results.

Regarding galactosan and mannosan, although very few laboratories reported their concentrations in SRM1649b, the results of this study were in good agreement with those previously reported for both techniques (Kirchgeorg et al. 2014; Louchouarn et al. 2009; Verlhac 2013).

Furthermore, despite the discrepancies in the standard material, it is important to note that no significant differences were observed between ultrasound and ASE extraction techniques when spiked filters or real atmospheric samples were analyzed in this study (Fig. 1, Table 2). Therefore, it can be concluded that the particular matrix of this material may be responsible for these discrepancies, as previously suggested by other authors.

The SRM 1649b matrix is complex and aged, and may contain strongly bound or trapped anhydrosugars that are released differently depending on the conditions of extraction, storage temperature, and sample preparation procedures. However, no definitive explanation has yet been established, and the true levoglucosan concentration in SRM 1649b remains uncertain.

The significant variability observed with SRM 1649b, likely due to storage conditions and matrix aging, contrasts sharply with the consistent results obtained for SRM 2787 in this study. These findings reinforce the reliability of SRM 2787 as a robust quality control standard and support the transition toward using newer-generation certified reference materials to ensure analytical consistency in biomass burning monitoring.

## Conclusions

An optimized and validated analytical framework based on ASE-derivatization-GC/MS was successfully established for the precise determination of levoglucosan, galactosan and mannosan in atmospheric particulate matter. The use of DCM/MeOH (2:1, v/v) proved to be the most efficient extraction solvent system, providing high recoveries for polar anhydrosugars while also enabling the simultaneous extraction of other key pollutants, like PAHs. Ultrasound-assisted extraction and accelerated solvent extraction (ASE) provided comparable recoveries for spiked filters and real atmospheric samples. However, ASE was selected as the preferred technique due to its higher precision, reduced handling and automation, making it ideal for large-scale environmental monitoring.

A critical finding for international quality assurance is the distinct performance of Certified Reference Materials. While SRM 2787 (Fine particulate matter) demonstrated high reliability and accuracy for routine method validation, SRM 1649b revealed significant variability in levoglucosan yields driven by matrix aging and pre-treatment conditions. This underscores the need for cautious selection of reference standards in atmospheric chemistry studies to avoid confounding matrix interferences with true environmental variations.

The comprehensive bottom-up uncertainty assessment revealed that overall methodology variance is heavily dominated by the chromatographic calibration step. Utilizing internal standardization combined with sedoheptulosan as a surrogate successfully minimized matrix effects for ambient air filters. Consequently, future efforts to improve data quality in source apportionment models should prioritize refined calibration strategies, such as weighted regression models, over further adjustments to sampling protocols.

The developed method offers a robust, high-throughput, and regulatory-compliant tool for air quality management networks. By refining the quantification of biomass burning tracers, this work directly supports the implementation of strict international air quality standards, and enhances the accuracy of source apportionment models used to evaluate the impact of biomass emissions on public health and regional climate.

## Supplementary information

Below is the link to the electronic supplementary material.

ESM 1 (DOCX 23.8 KB)

## Data Availability

All data supporting the findings of this study are available within the paper and its supplementary information. Additional raw data are available from the corresponding author upon reasonable request.
