# Supplementary material for: Tracking biomass burning markers in atmospheric aerosols: Robust quantification of anhydrosugars and reference material evaluation
Source: Environ Sci Pollut Res Int. 2026 Aug 11;33(26):13367–77. doi: 10.1007/s11356-026-38136-6 (PMC13503485; doi:10.1007/s11356-026-38136-6)
Supplement: Supplementary file 1 [file 11356_2026_38136_MOESM1_ESM.docx]

Supplementary information

**Tracking biomass burning markers in atmospheric aerosols: Robust quantification of anhydrosugars and reference material evaluation**

Journal: Environmental Science & Pollution Research

Rosa Pérez-Pastor, Susana García-Alonso, María Leco, Óscar Pindado

CIEMAT-Chromatography Unit

Avenida Complutense, 40 28040 Madrid, Spain

Email: [rosa.perez@ciemat.es](mailto:rosa.perez@ciemat.es)

1. **Uncertainty of the preparation of the stock solution from pure substances**

$C_{\mathrm{stk}}=\frac{1000 m P}{V}$ (mg L^-1^)

The purity of the standards appears in the certificate as > 99% (0.99±0.01). As there is not additional information about the uncertainty, we assumed a rectangular distribution

$$u\left( P \right)=\frac{0.01}{\sqrt{3}}=0.0058$$

The uncertainty associated to the mass of compound depends on the performance of the analytical balance. This was calculated following an internal quality protocol as 7.5 x 10 ^-5^ g.

The volume has three main influences: calibration, repeatability and temperature effect.

Calibration. The manufacturer set the flask volume as 10 ml ± 0.025 (20ºC). Standard uncertainty is calculated assuming a triangular distribution:

$$\frac{0.025 mL}{\sqrt{6}}=0.01mL$$

Uncertainty derived from repeatability in the filling of the flask was evaluated as a standard uncertainty performing a set of 10 experiments by filling and weighting a flask. A standard deviation of 0.006 mL was achieved.

According to the manufacturer the flask was calibrated at 20ºC. The temperature in the laboratory can vary ± 4ºC. The contribution to uncertainty of the variation of temperature can be evaluated through the estimation of the range of temperatures and the volume coefficient of expansion. The expansion volume of liquid is bigger than the flask, so only is needed to consider the first one.

The expansion coefficient of the methanol volume is 1.4 x 10^-3^ ºC^-1^, which leads to a volume variation of:

± (10 x 4 x 1.4 x 10^-3^) =± 0.06 mL

Standard uncertainty is calculated assuming a rectangular distribution for the temperature variation:

$$\frac{0.06 mL}{\sqrt{3}}=0.03 mL$$

These three contributions are combined to provide standard uncertainty u (V) of volume:

$$u\left( V \right)=\sqrt{{0.01}^{2}+{0.006}^{2}+{0.03}^{2}}=0.036 mL$$

For intermediate values (1 mg anhydrosugar), the combined standard uncertainty for the gravimetric solution is calculated according to the above equation.

$$C_{anh}=\frac{1000 x 1 x 0.99}{10}=99 mg L^{-1}$$

$$\frac{u(C_{anh})}{C_{anh}}=\sqrt{{0.00586}^{2}+{0.000075}^{2}{+0.036}^{2}}=0.036$$

$$u\left( C_{anh} \right)=C_{anh} x 0,036=99 mg L^{-1}x 0,03=3.6 mg L^{-1}$$

2. **Uncertainty due to the dilution of the original stock solution.**

During dilutions, the temperature will remain constant, so, for this calculations, only tolerance of the syringes and repeatability were considered.

$$C_{dil}=C_{stk}\frac{V_{stk}}{V_{dil}}$$

The uncertainty is calculated according to:

$$\left( \frac{u_{Cdil}}{C_{dil}} \right)^{2}=\left( \frac{u_{Cstk}}{C_{stk}} \right)^{2}+\left( \frac{u_{Vf}}{V_{f}} \right)^{2}+\left( \frac{u_{V0}}{V_{0}} \right)^{2}$$

Being V_0_ the volume of the stock solution used, and V_f_, the volume final of the dilution.

Table represents the uncertainties associated to the syringes employed to prepare calibration solutions.

The tolerance specified by the manufacturer is ±1% of the nominal volume

$$u_{tol}=tol/\sqrt{3}$$

Repeatability was calculated from five replicate weighing (f=1.4) of syringes filled with methanol, that included volumes of 1000, 500, 250, and 100 µL. f is an statistical factor which depends on the number of experimental replicates, employed to estimate the repeatability standard deviation from the experimental range.

$$u_{rep}=f\frac{s_{rep}}{\sqrt{n}}$$

| Syringe vol  (mL) | Tolerance  (mL) | $\frac{u_{tol}}{V}$ | Repeatability | $\frac{u_{rep}}{V}$ | $\left( \frac{u_{rep}}{V} \right)^{2}$ |
| --- | --- | --- | --- | --- | --- |
| 1 | 0.01 | 0.0058 | 0.008 | 0.005 | 2.5 E^-5^ |
| 0.5 | 0.005 | 0.0025 | 0.003 | 0.004 | 1.6 E^-5^ |
| 0.25 | 0.0025 | 0.0008 | 0.001 | 0.0025 | 6.2 E^-6^ |
| 0.1 | 0.001 | 0.0004 | 0.0005 | 0.003 | 9.0 E^-6^ |

| Syringe vol  (mL) | $\frac{u_{tol}}{V}$ | $\left( \frac{u_{tol}}{V} \right)^{2}$ | $\left( \frac{u_{V}}{V} \right)^{2}$ |
| --- | --- | --- | --- |
| 1 | 5.8E-3 | 3.3 E^-5^ | 5.8 E^-5^ |
| 0.5 | 5.8E-3 | 3.3 E^-5^ | 4.9 E^-5^ |
| 0.25 | 5.8E-3 | 3.3 E^-5^ | 3.9 E^-5^ |
| 0.1 | 5.8E-3 | 3.3 E^-5^ | 4.2 E^-5^ |

Uncertainty associated with calibration solutions

To obtain uncertainties of each step of dilution, a factor was applied from the used syringes (Pindado and Pérez Pastor, 2014):

F=V_f_/V_0_

| Dis | Conc  (ug/mL) | Stock sol  µg/ml | F | V_0_ | V_F_ | $\left( \frac{u_{F}}{F} \right)^{2}$ |
| --- | --- | --- | --- | --- | --- | --- |
| 1 | 30 | 100 | 1/3 | 300 | 1000 | 1.1 E^-4^ |
| 2 | 20 | 100 | 1/5 | 200 | 1000 | 9.7 E^-5^ |
| 3 | 10 | 100 | 1/10 | 100 | 1000 | 1.0 E^-4^ |
| 4 | 5 | 20 | 1/4 | 250 | 1000 | 9.7 E^-5^ |
| 5 | 2 | 10 | 1/5 | 200 | 1000 | 1.0 E^-4^ |

3. **Uncertainty due to the extract volume:**

Uncertainty in the final extract volume (1 mL) arises from:

a) Syringe tolerance (u_t_), which was calculated through a triangular distribution:

$\left( \frac{\left( tol/\sqrt{3} \right)}{V} \right)^{2}=\left( \frac{\left( 0.01/\sqrt{3} \right)}{1} \right)^{2}$=3.3 E^-5^

b) Temperature: Same calculation than section 1. ESM

$$\left( \frac{\left( V T coef exp/\sqrt{3} \right)}{T} \right)^{2}=\left( \frac{(1 x 4 x 1.4 x {10}^{-3}/\sqrt{3})}{4} \right)^{2}=6.5 E^{-7}$$

c) Repeatability (ur) which is calculated as standard deviation from ten weighings of the filled syringe with methanol

$$\left( \frac{0.008}{1} \right)^{2}=6.4 E^{-5}$$

$$\left( \frac{u_{Vf}}{V_{f}} \right)^{2}=5.8 E^{-5}$$
